# Insulin-like Growth Factor 2 Promotes Tissue-Specific Cell Growth, Proliferation and Survival during Development of *Helicoverpa armigera*

**DOI:** 10.3390/cells11111799

**Published:** 2022-05-31

**Authors:** Yu-Meng Zhao, Xiao-Pei Wang, Ke-Yan Jin, Du-Juan Dong, Tobias Reiff, Xiao-Fan Zhao

**Affiliations:** 1 Shandong Provincial Key Laboratory of Animal Cells and Developmental Biology, School of Life Sciences, Shandong University, Qingdao 266237, China; zhaoyumeng8033@163.com (Y.-M.Z.); xiaopei_w9507@163.com (X.-P.W.); 17853509601@163.com (K.-Y.J.); dongdj@sdu.edu.cn (D.-J.D.); 2 Institute of Genetics, Heinrich-Heine-University, 40225 Düsseldorf, Germany

**Keywords:** IGF, cell proliferation, imaginal midgut, imaginal fat body, 20-hydroxyecdysone

## Abstract

During development, cells constantly undergo fate choices by differentiating, proliferating, and dying as part of tissue remodeling. However, we only begin to understand the mechanisms of these different fate choices. Here, we took the lepidopteran insect *Helicoverpa armigera*, the cotton bollworm, as a model to reveal that insulin-like growth factor 2 (IGF-2-like) prevented cell death by promoting cell growth and proliferation. Tissue remodeling occurs during insect metamorphosis from larva to adult under regulation by 20-hydroxyecdysone (20E), a steroid hormone. An unknown insulin-like peptide in the genome of *H. armigera* was identified as IGF-2-like by sequence analysis using human IGFs. The expression of *Igf-2-like* was upregulated by 20E. IGF-2-like was localized in the imaginal midgut during tissue remodeling, but not in larval midgut that located nearby. IGF-2-like spread through the fat body during fat body remodeling. Cell proliferation was detected in the imaginal midgut and some fat body cells expressing IGF-2-like. Apoptosis was detected in the larval midgut and some fat body cells that did not express IGF-2-like, suggesting the IGF-2-like was required for cell survival, and IGF-2-like and apoptosis were exclusive, pointing to a survival requirement. Knockdown of *Igf-2-like* resulted in repression of growth and proliferation of the imaginal midgut and fat body. Our results suggested that IGF-2-like promotes cell growth and proliferation in imaginal tissues, promoting cell death avoidance and survival of imaginal cells during tissue remodeling. It will be interesting to determine whether the mechanism of action of steroid hormones on insulin growth factors is conserved in other species.

## 1. Introduction

Insulin-like growth factors (IGFs) belong to the insulin-like peptide (ILP) superfamily. ILPs are divided into insulin, relaxin and IGF. All ILPs possess a signal peptide, B-chain, C-peptide and A-chain from the N-terminus to the C-terminus. However, the C-peptide is cleaved during the process of forming the mature peptide in insulin and relaxin, and thus insulin and relaxin exert their roles as heterodimers composed of an A-chain and a B-chain. In contrast, the C-peptide of IGFs is not cleaved, and thus IGFs exert their roles as single chains [1]. The main function of insulin is to promote the metabolism of glucose and lipids by binding to the insulin receptor (INSR). IGFs promote tissue growth by binding to the IGF receptor (IGFR), a receptor tyrosine kinase (RTK) for IGF-1, and the cation-independent mannose 6-phosphate (M6P) receptor for IGF-2 [2]. Relaxins mainly play roles in pregnancy and childbirth by binding to G protein-coupled receptors (GPCRs) [1,3].

There are ten ILPs in mammals, including two IGFs, IGF-1 and IGF-2 [4]. Mammalian IGFs are mainly produced by the liver and play important roles in embryonic and postpartum growth. IGF-1 maintains the steady growth of the body and promotes tissue growth [5]. IGF-2 acts as a fetal growth factor [6], playing an important role in the maintenance of neurogenesis in adult rats [7]. It has been reported that IGF-2 promotes glucose transport by interacting with INSR [8]. IGFs play important roles in humans and animals in physiological processes and cancers; however, despite four decades of research, many questions remain about how insulin-like bioactivities are controlled [9]. The identification and characterization of novel regulators of the IGF are an emerging area of cancer research [10].

The mechanisms of insulin/insulin-like growth factor signaling (IIS) have been well conserved during evolution in insects and mammals [11]. IGFs analogs are also found in invertebrates. There are 33 ILPs [12] in *Bombyx mori*. An IGF-like peptide (BIGFLP, NP_001138796, AB360450) in *B. mori* hemolymph, produced by the fat body at the pupa imago stage without cleavage, promotes the growth of imaginal-specific tissues in in vitro culture, but it does not promote growth of the fat body [13]. There are eight ILPs in *Drosophila melanogaster* [14]. *Drosophila* insulin-like peptide 6 (DILP6), produced in the fat body, is similar to mammalian IGF in terms of its structure and function [15]. It is highly expressed in the fat body at the end of third instar and in the pupa to imago stage. Mutation of DILP6 leads to a decrease in body weight [16]. DILP8 is secreted by many tissues in *Drosophila* to accurately control insect size and development [17]. DILP8 also acts on neurons to regulate feeding and metabolism [18]. Eight ILPs are highly expressed in *Helicoverpa armigera* during metamorphosis [19]; however, an IGF has not been identified and thus the roles of IGF in *H. armigera* are unclear.

In holometabolous insects, tissue remodeling occurs during metamorphosis, in which the larval tissues die by programmed cell death and imaginal tissues form by cell proliferation. For example, the midgut, the main digestive organ of insects, undergoes remodeling during metamorphosis. During this process, the larval midgut begins autophagy, followed by apoptosis, whereas the imaginal midgut grows and proliferates to form the adult midgut. The larval midgut is inside and the imaginal midgut is outside, as shown by cross-sectional slides [20]; however, the mechanism by which the larval midgut dies, but the nearby imaginal midgut grows and proliferates, is unclear.

Similarly, the fat body, an important nutritional and metabolic organ of insects with functions like those of the mammalian liver and adipocytes [21], also undergoes remodeling during metamorphosis [22]. However, the larval fat body and imaginal fat body have never been distinguished during fat body remodeling because they are not separated into inside and outside layers like midgut remodeling during metamorphosis. Autophagy and apoptosis are observed in the fat body during its remodeling [23]; however, some fat cells exist until eclosion to provide energy for imago development [22]. The surviving fat body cells regenerate into imaginal fat body cells [24], acting as imaginal progenitor cells [25]. However, the mechanism by which some fat body cells survive and proliferate, while other fat body cells die, and the marker of imaginal fat body progenitor cells, are currently unclear.

In the present study, we used the lepidopteran insect *H. armigera*, the cotton bollworm, as a model to study these questions. We identified IGF-2-like in *H. armigera*, examined its expression and location in tissues during metamorphosis, and detected 20-hydroxyecdysone (20E) regulation of IGF-2-like. IGF-2-like was localized in the imaginal midgut and in some fat body cells during metamorphosis. Knockdown of *Igf-2-like* led to blockade of imaginal midgut and fat body formation. We concluded that the expression of IGF-2-like in the imaginal midgut and fat body promotes their growth and proliferation.

## 2. Experimental Materials and Methods

### 2.1. Experimental Animal

*H. armigera* were fed with artificial food in our laboratory according to a previously published method [26]. The living environment temperature was 25–27 °C, the humidity was 40–60%, and the daily light duration was 14 h.

### 2.2. Bioinformatic Analysis

The full-length sequences of the genes were obtained from NCBI (https://www.ncbi.nlm.nih.gov/ (accessed on 2 May 2022)). The molecular weight and isoelectric point were analyzed using the ExPASY website ((https://web.expasy.org/compute_pi) (accessed on 2 May 2022)). The phylogenetic tree was built using MAGE 5.0, and the sequence alignment was performed using DNAMAN ((https://www.lynnon.com/) (accessed on 2 May 2022)).

### 2.3. Gene Cloning

Respectively, 100 mg of the dorsal epidermis, midgut, fat body and brain were collected from different stages. The tissues were rinsed in 1 × phosphate-buffered saline (PBS) (140 mm NaCI, 2.7 mm KCI, 10 mm Na_2_HPO_4_, 1.8 mm KH_2_PO_4_), and then added to 1.5 mL tubes filled with Trizol (O10820, TransGen Biotech, Beijing, China) for homogenization. The RNA was extracted according to the instructions for Trizol. The RNA was used directly or stored at −20 °C.

### 2.4. Quantitative Real-Time Reverse Transcription PCR (qRT-PCR)

From the purified insect RNA, cDNA was obtained using 5× all-in-one RT MasterMix reverse transcription Kit (G492, Abm, Richmond, BC, Canada). The cDNA was used as the template in the quantitative real-time PCR (qPCR) step of the qRT-PCR protocol. The 10 μL qPCR reaction comprised 1 μL (600 ng/μL) of cDNA template, 2 μL (1 μM) of forward primers, 2 μL (1 μM) of reverse primers, and 5 μL of TransStart Tip Green qPCR Supermix (291829AX, Aidlab, Beijing, China). The qPCR reaction comprised pre-denaturation for 10 min at 95 °C, followed by 40 cycles of denaturation for 10 s at 95 °C and annealing for 1 min at 60 °C. The expression difference was calculated using the cycle threshold (Ct) value given by the instrument. The calculation formula was 2^−^^△△ Ct^ = 2^–(^^△ Ct experimental group −^^△ Ct control group)^, △ Ct ^experimental group^ = Ct ^experimental group−*Actb* mean value^ and △ Ct ^control group^ = Ct ^control group−*Actb* mean value^, in which *Actb* encodes β-actin.

### 2.5. 20E Stimulation in Larvae

The 20E was dissolved at 10 mg/mL (20 mm) in dimethyl sulfoxide (DMSO) and then diluted to different concentrations (20–100 ng/μL) with filtered 1 × PBS. We injected 5 μL of 20E into the body of 6th instar 6 h (6th-6 h) larva for 12 h, with an equal amount of diluted DMSO as the control. The time gradient hormone stimulation was set by injecting 5 μL 20E (100 ng/μL) into the 6th-6 h larva for 3–24 h.

### 2.6. Preparation of Rabbit Polyclonal Antibody

The full-length cDNA of IGF-2-like was amplified using the primers Exp-Igf-2-like-F and Exp-Igf-2-like-R (Supplementary Table S1), and inserted into the plasmid pET-30a (+) (69909–3, Novagen, Madison, WI, USA). The plasmid construct was transformed into *Escherichia coli* (BL21-DE) 645 (c1400, Solarbio Life Sciences, Beijing, China). IGF-2-like was expressed in the inclusion bodies. IGF-2-like was denatured and purified using a smart Ni-NTA beads 6FF column (SA005500, Smart-Lifesciences, Changzhou, China). After renaturation, 1 mg/mL protein was mixed with complete adjuvant by 1:1 volume ratio and injected subcutaneously into New Zealand white rabbits. After 21 days, the renatured protein was mixed with incomplete adjuvant and injected directly. Seven days later, the blood was taken, and the supernatant was centrifuged to obtain anti-IGF-2-like antibody.

### 2.7. Western Blotting

Respectively, 100 mg of the dorsal epidermis, midgut, and fat body, and 1 mg of brain, were dissected, washed with 1 × PBS, and homogenized in 40 mm Tris-HCl with 1 mm Phenylmethylsulfonyl fluoride (PMSF) (pH 8.0). The cell debris was removed after high-speed centrifugation. Then, 50 μL of hemolymph was added with 5 μL of 10 × EDTA 2K anticoagulant (G0280, Solarbio Life Sciences, Beijing, China), centrifuged at 3000× *g* to remove blood cells, and the supernatant was diluted with 1 × PBS (1:8). Next, 50 μg of protein from each sample were separated using 12.5–15% sodium dodecyl sulfate polyacrylamide gel electrophoresis (SDS-PAGE), transferred onto a nitrocellulose membrane, and blocked with 5% fat-free powdered milk in TBS (10 mM Tris-HCl, 150 mm NaCl, pH 7.5) for 1 h. The primary antibodies (IGF-2-like serum 1:100 dilution, beta actin (ALP73405.1) rabbit mAb (AC026), ABclonal, Wuhan, China) were added and incubated overnight at 4 °C. The secondary antibodies (Goat anti rabbit IgG/alkaline phosphatase labeled (Zb-2308, Zhongshanjinqiao, Beijing, China)) were then added and incubated at room temperature for 2 h. TBST (0.02% Tween20 in TBS) was used to wash the membrane three times for 10 min each time. The signals were detected by incubating the membrane in a chromogenic solution (TBS containing 5% p-nitro-blue tetrazolium chloride (6578-06-9, NBT, Sangon Biotech, Shanghai, China) and 5% 5-bromo-4-chloro-3-indolyl phosphate (6578-06-9, BCIP, Sangon Biotech)).

### 2.8. RNAi of Larvae by Feeding E. coli (HT115/DE3)

We selected a fragment of the target gene for PCR amplification and inserted it into vector pPD129.36 (L4440), which was gifted by Dr. Marek Jindra (Biology Center, Czech Academy of Sciences, Institute of Entomology, Ceske Budejovice, Czech Republic). Plasmids from positive clones screened by PCR were extracted and transformed into *E. coli* HT115 cells, which were also provided by Dr. Jindra. Positive clones were screened by PCR and cultured in medium at 37 °C overnight. Overnight strains were added into medium (pH 7.4, 0.5 g yeast extract, 1 g tryptone, 1 g NaCl, 100 mL ddH_2_O) with 50 μL of ampicillin (25 mg/mL) and 6.25 μL of tetracycline (25 mg/mL) at a ratio of 1:100. After shaking culture for 3 h, Isopropyl β-D-1-thiogalactopyranoside (IPTG, 0.5 mm) was added to the medium and shaken continuously for 4 h. The bacteria were collected by centrifugation and then diluted with PBS. Fresh HT115 cells that expressed the dsRNA diluted in PBS, were dropped onto fodder that was cut into 1 cm × 1 cm × 0.2 cm pieces and fed to larvae according to a previously published method [27]. The food was replaced every day up to the 6th-72 h stage. Control groups were fed with fodder containing *dsGFP*-expressing HT115 cells. Total RNA was extracted from different tissues after *H. armigera* stopped feeding.

### 2.9. RNAi by Injection

*dsIgf-2-like* and *dsGFP* were synthesized using Igf-2-like-RNAiF, Igf-2-like-RNAiR, GFP-RNAiF, GFP-RNAiR (Supplementary Table S1) according to the synthetic double-chain method described by Chen et al. [28]. We injected 5 μL of 0.4 μg/μL dsRNA into the body of 6th-72 h larva. Three injections were given every 24 h, with 30 larvae in each group.

### 2.10. Immunofluorescence

The midgut and fat body were fixed using 4% paraformaldehyde at 4 °C overnight. Samples were sent to Servicebio (Wuhan, China) for hematoxylin and eosin (HE) staining and immunofluorescence localization. The primary antibodies were rabbit serum anti-IGF-2-like (1:50), rabbit polyclonal antibody against Caspase-3 (CASP3) (1:200) (GB11532, Servicebio, Wuhan, China), and rabbit anti-phospho-histone H3 (Ser10) antibody (1:200) (9701, Cell Signaling Technology, Danvers, MA, USA). The fluorescent-labeled secondary antibodies were Alexa fluor 488-conjugated goat anti-rabbit IgG (H+L) (GB25303, Servicebio, Wuhan, China) and Cy3-conjugated goat anti-rabbit IgG (H+L) (GB21303, Servicebio, Wuhan, China). An identical slide was double-immunoassayed to co-localized IGF-2-like and phospho-histone H3 or CASP3 by a sequential staining method. The slide was first stained to show phospho-histone H3 or CASP3 by the corresponding antibodies, rabbit anti-phospho-histone H3 (Ser10) antibody, or rabbit polyclonal antibody against CASP3 overnight at 4 °C. After being well-washed three times in PBS (pH 7.4) for five min each time, the slide was stained by Alexa fluor 488-conjugated goat anti-rabbit IgG (H+L) for 50 min at room temperature. After being well-washed three times in PBS (pH 7.4) for five min each time to remove the primary antibodies, the slide was examined for fluorescence, and then stained to show IGF-2-like by the corresponding antibodies, rabbit serum anti-IGF-2-like and Cy3-conjugated goat anti-rabbit IgG (H+L). The slides were then stained with 4′,6-diamidino-2-phenylindole (DAPI) (G1012, Servicebio, Wuhan, China) for 10 min at room temperature in the dark. The slides were viewed with a fluorescence microscope (DAPI excitation wavelength 330–380 nm, emission wavelength 420 nm, blue light; Alexa 488 excitation wavelength 465–495 nm, emission wavelength 515–555 nm, green light; CY3 excitation wavelength 510–560 nm, emission wavelength 590 nm, red light).

### 2.11. Statistical Analysis

All the experiments were repeated three times. qRT-PCR met the requirements of three biological and three technical repetitions. Significant differences between two samples were tested using a two-tailed Student’s *t*-test. * *p* < 0.05, ** *p* < 0.01, and *** *p* < 0.001 present significant differences.

## 3. Results

### 3.1. Identification of IGF-2-Like of H. armigera

**Figure 1:**
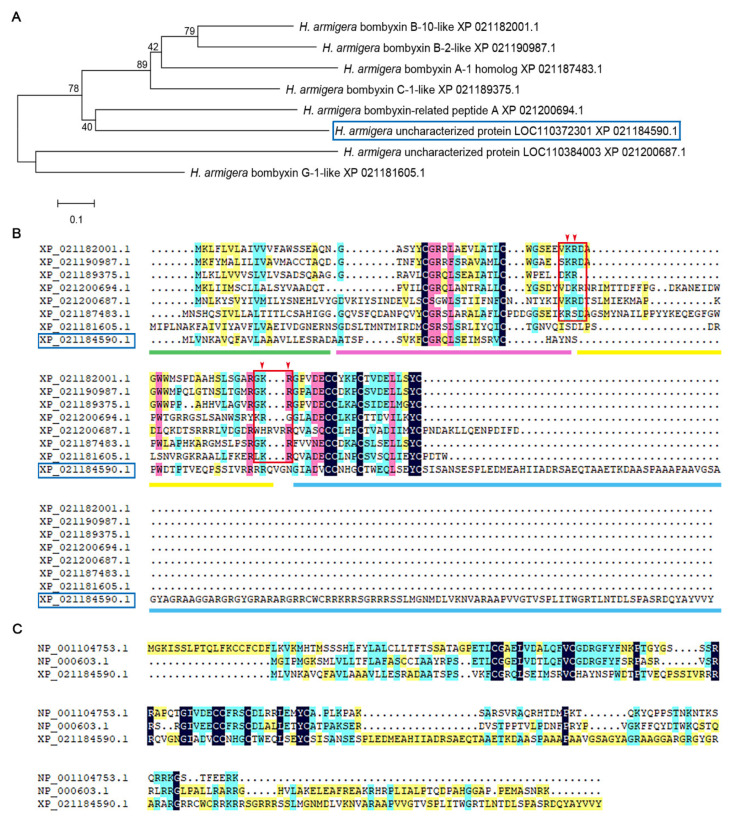
Identification of IGF-2-like of *H. armigera*. (**A**) A phylogenetic tree of ILPs in *H. armigera* analyzed by MAGE5. The blue box indicates IGF-2-like. (**B**) Sequence alignment of the ILPs of *H. armigera*. The red box marks the potential splicing sites (the double alkaline amino acids) at both ends of the C peptide. Green underline: signal peptide; pink underline: B chain; yellow underline: C peptide; blue underline: A chain. (**C**) Comparison of IGF-2-like with IGF-1 and IGF-2 of *H. sapiens*.

A total of 8 ILPs were found in the genome of *H. armigera* using 33 ILPs from *B. mori* [12], 10 ILPs from *Homo sapiens* [4], and 8 ILPs from *D. melanogaster* [14] as query sequences (Figure 1A). To identify the IGFs in *H. armigera*, the sequences of eight ILPs were aligned using DNAMAN to find the potential cleavage sites (the double alkaline amino acids, KR, KK, RR and RK) at both ends of the C peptide, according to a previous study [3]. The alignment showed that KR was conserved at both sides of C peptide in six ILPs. The *Bombyx* ILP, bombyxin G-1-like (XP_021181605.1), had KR at the second cleavage site of C peptide, and another ILP, from *H. armigera*, uncharacterized protein LOC11037230 (XP_021184590.1), had no double alkaline amino acids KR at both sides of the C peptide, although an RR site was nearby (Figure 1B). The sequence of uncharacterized protein LOC11037230 (XP_021184590.1) had 32.58% identity with *H. sapiens* IGF-2, which was higher than the 28.92% identity with *H. sapiens* IGF-1, and thus we named it as IGF-2-like (Figure 1C). The phylogenetic tree showed that *H. armigera* IGF-2-like clustered with ILPs of *B. mori* (XP 012552554.1), *H. sapiens* (INSL5 NP 005469.2), and *D. melanogaster* ILP8 (NP 648949.2) (Supplementary Figure S1).

**Figure 2:**
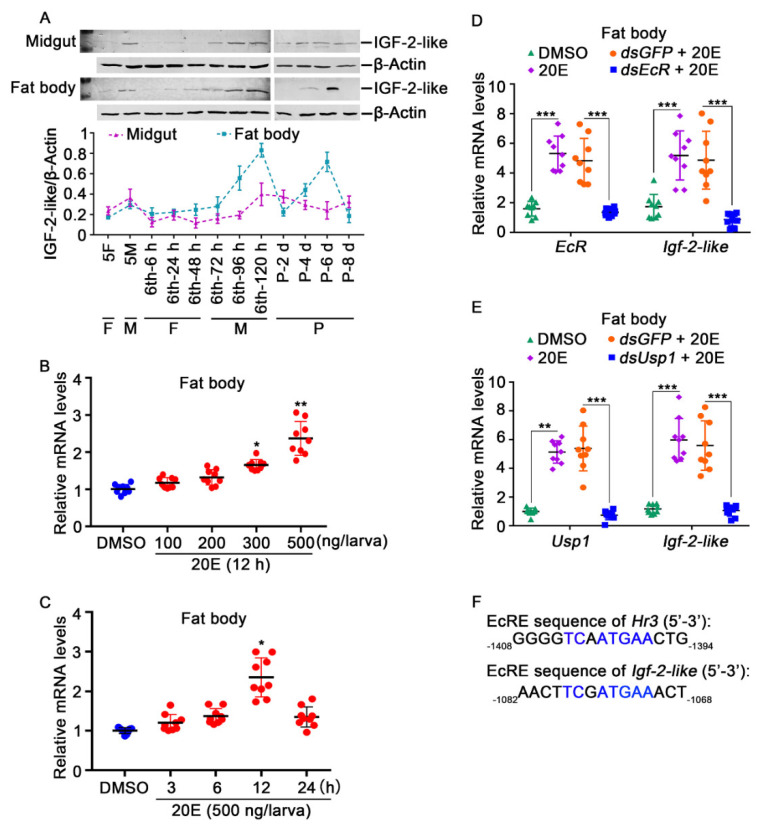
*Igf-2-like* mRNA expression and 20E stimulation. (**A**) The protein levels of IGF-2-like in the midgut and fat body at different stages. β-ACTB was used as an internal reference protein. The concentration of the gel was 12.5%. Image J was used to calculate the ratio of IGF-2-like and β-ACTB band densities. All the experiments comprised three biological replicates. The error bar indicates the mean ± SD. 5F: the 5th instar feeding stage; 5M: the 5th molting stage; 6th-6 h, 6th-24 h, 6th-48 h, 6th-72 h, 6th-96 h and 6th-120 h correspond to different stages of the 6th instar; P-2 d, P-4 d, P-6 d and P-8 d correspond to different pupal stages. F: feeding stage; M: molting stage; MM: metamorphic molting stage; P: pupal stage. (**B**) The expression of *Igf-2-like* in the fat body after 12 h of stimulation with various concentrations of 20E. (**C**) Different time gradients were used to detect the regulation of *Igf-2-like* by 20E (500 ng/larva), and the same amount of DMSO was used as a negative control. Three biological replicates were performed for each experiment, and each biological replicate included three technical replicates, represented by dots. (**D**,**E**) *EcR* and *Usp1* knockdown in the fat body using *dsEcR* or *dsUsp1* (2 μg/larva), followed by stimulation with 20E (500 ng/larva) for 12 h to detect *Igf-2-like* expression, qRT-PCR experiments were carried out using three biological and three technical repetitions. The error bar indicates the mean ± SD. Student’s *t*-test analysis indicates the significant differences (* *p* < 0.05, ** *p*< 0.01, *** *p* < 0.001). (**F**) Alignment of the EcRE in the 5′-upstream region of *Hr3* and *Igf-2-like*.

The expression profiles of IGF-2-like were examined using rabbit polyclonal antibodies against *H. armigera* IGF-2-like to show its tissue and developmental stage characteristics. The antibody recognized IGF-2-like from the 6th-96 h larval fat body specifically. The molecular weight of IGF-2-like was 30 kDa, with no cleaved fragment (Supplementary Figure S2), which was 5 kDa larger than the predicted molecular weight of IGF-2-like (24.3 kDa), possibly because of the higher content of lysine and arginine in IGF-2-like (13.7%). The specificity of the antibody was confirmed using IGF-2-like-His (IGF-2-like 30 kDa plus 6 kDa His tag) expressed in *E. coli* (Supplementary Figure S3A). The Western blotting results showed that little IGF-2-like was detected from hemolymph after the removal of blood cells (Supplementary Figure S3B). However, IGF-2-like was highly expressed in the midgut and fat body at the metamorphic stage (Figure 2A). IGF-2-like was also expressed in the epidermis and brain, with high expression at the feeding stage (Supplementary Figure S4). The high expression of IGF-2-like in the midgut and fat body during metamorphosis suggested that it was upregulated by 20E, because 20E has a high titer during metamorphosis [29].

Therefore, a 20E stimulation experiment was carried out to examine the regulation of 20E on *Igf-2-like*. The results showed that the expression of *Igf-2-like* was significantly upregulated in the fat body by 300–500 ng 20E injection into the hemolymph, and the upregulation trend correlated positively with the 20E concentration (Figure 2B) and time (Figure 2C), suggesting that *Igf-2-like* was upregulated by 20E. We knocked down *EcR* (encoding the ecdysone receptor (20E receptor)) and *Usp1* (encoding ultraspiracle protein, the heterodimer USP1 of 20E) in the fat body to confirm that 20E upregulated *Igf-2-like* expression. The results showed that, after *EcR* and *Usp1* knockdown, *Igf-2-like* expression was reduced (Figure 2D, E). By analyzing the *Igf-2-like* promoter sequence, an ecdysone-response element (EcRE) that could be bound by EcR [30,31,32] was predicted in the upstream sequence of *Igf-2-like* (Figure 2F). These data suggested 20E upregulated *Igf-2-like* expression via EcR.

### 3.2. IGF-2-like Was Localized in the Imaginal Midgut and in Some Fat Body Cells during Metamorphosis

**Figure 3:**
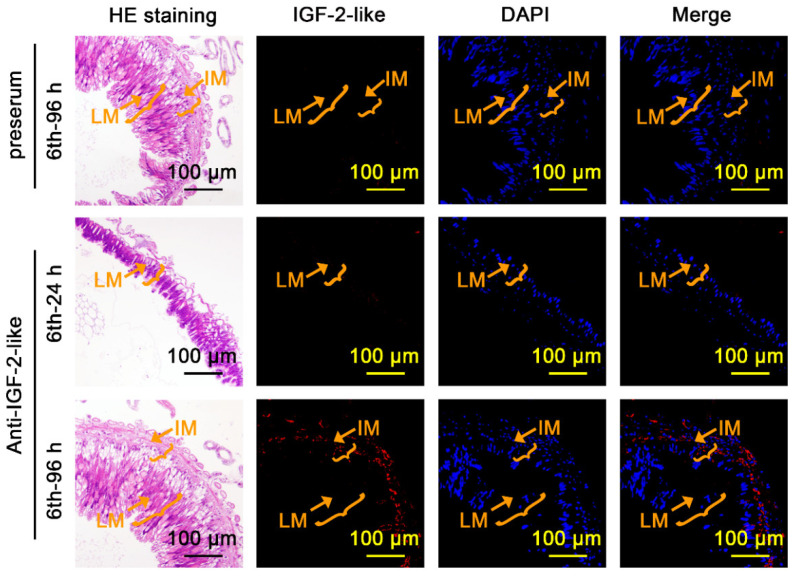
IGF-2-like was localized in the imaginal midgut at the metamorphic stage. HE staining showing the midgut structure after immunofluorescence staining. LM: larval midgut; IM: imaginal midgut; red fluorescence: IGF-2-like detected by anti-IGF-2-like rabbit polyclonal antibodies and Cy3-conjugated goat anti-rabbit IgG (H+L) antibodies; blue fluorescence: DAPI. Preserum was used as the negative control. Merge: IGF-2-like and DAPI.

To address the function of IGF-2-like in the larval midgut or imaginal midgut during metamorphosis, the localization of IGF-2-like in the larval midgut and imaginal midgut was observed using immunohistochemistry. The midgut of the 6th-96 h larvae at metamorphosis contains an inner larval midgut and an outer imaginal midgut. No IGF-2-like was detected using preserum, a negative control of the antibody against IGF-2-like (anti-IGF-2-like). The midgut of 6th-24 h larvae at the feeding stage appeared as a single cell layer, and no IGF-2-like was detected using the anti-IGF-2-like antibody. However, the imaginal midgut of the 6th-96 h midgut was stained using the anti-IGF-2-like antibody (Figure 3). The results showed that IGF-2-like was localized to the imaginal midgut.

**Figure 4:**
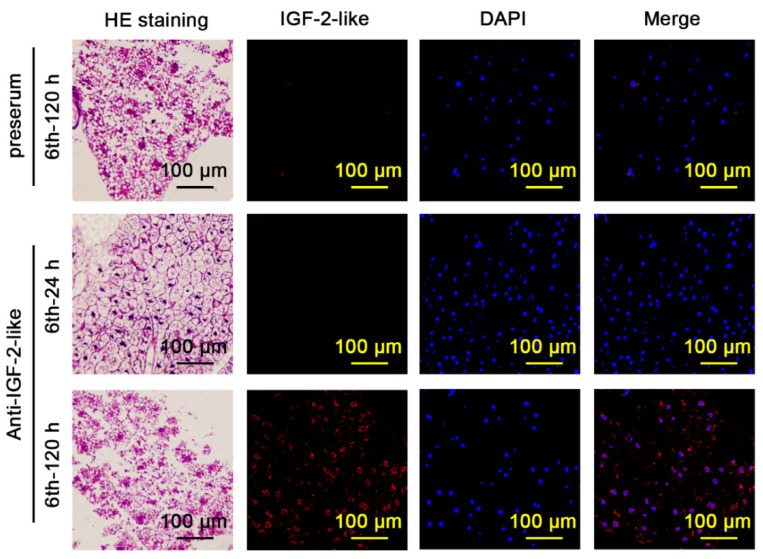
IGF-2-like was localized in imaginal fat body. HE staining showing the fat body structure of corresponding immunofluorescence tissue. Red fluorescence: IGF-2-like detected by anti-IGF-2-like rabbit polyclonal antibodies and Cy3-conjugated goat anti-rabbit IgG (H+L) antibodies. Blue fluorescence: DAPI. Preserum was used as the negative control. Merge: IGF-2-like and DAPI.

The fat body of 6th-120 h larvae appeared loose and decomposed. The negative control preserum did not detect IGF-2-like in the fat body of 6th-120 h larvae. The fat body of 6th-24 h larvae was intact. The anti-IGF-2-like antibody also did not detect IGF-2-like in the fat body of 6th-24 h larvae. However, the anti-IGF-2-like antibody detected IGF-2-like in the fat body of 6th-120 h larvae, in which it was spread throughout the whole loosened fat body (Figure 4). The number of nuclei in the IGF-2-like-positive fat body stained by DAPI was significantly lower than that in the fat body of 6th-24 h larvae (Supplementary Figure S5), suggesting that some fat body cells died, whereas the cells expressing IGF-2-like survived.

**Figure 5:**
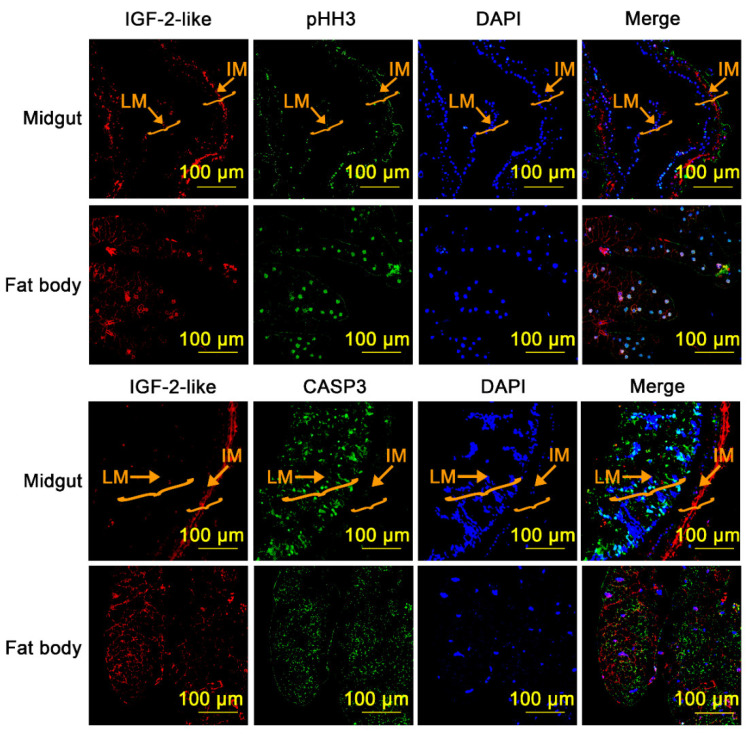
Immunofluorescence to show the tissue localization of IGF-2-like, pHH3, and CASP3. The midgut and fat body at 6th-96 h were used for immunofluorescence localization. An identical slide was double-immunoassayed to co-localized IGF-2-like and phospho-histone H3 or CASP3 by a sequential staining method. Red fluorescence represents IGF-2-like. The green fluorescence in above panels represents pHH3. The green fluorescence in below panels represents CASP3. LM: larval midgut. IM: imaginal midgut.

Histone H3 phosphorylation on serine-10 is unique to mitosis, and phosphorylated histone H3 (pHH3) is a marker of proliferation [33]. CASP3 is a marker of apoptosis [34]. We performed immunofluorescence to localize pHH3 or CASP3 in the 6th-96 h midgut and fat body, respectively, to detect cell proliferation and apoptosis. The results showed that some pHH3 was localized in the imaginal midgut, co-localized with IGF-2-like. Similarly, pHH3 was detected in the fat body, again co-localized with IGF-2-like. In contrast, CASP3 signals were detected in the larval midgut, where IGF-2-like was absent, but not from the imaginal midgut, where IGF-2-like was located. In the fat body, the CASP3 signal was detected in small pieces, which might represent the dying and decomposing fat body cells, whereas IGF-2-like was detected in other fat body cells that lacked the CASP3 signal (Figure 5). In the midgut and fat body of 6th-24 h larvae, no fluorescence signals of IGF-2-like, pHH3, or CASP3 were detected by the antibodies (Supplementary Figure S6). These data suggested that IGF-2-like functions to maintain imaginal midgut and fat body cell proliferation and prevent them from undergoing apoptosis during metamorphosis.

### 3.3. Knockdown of Igf-2-like Caused Repression of the Imaginal Midgut and Fat Body Formation

**Figure 6:**
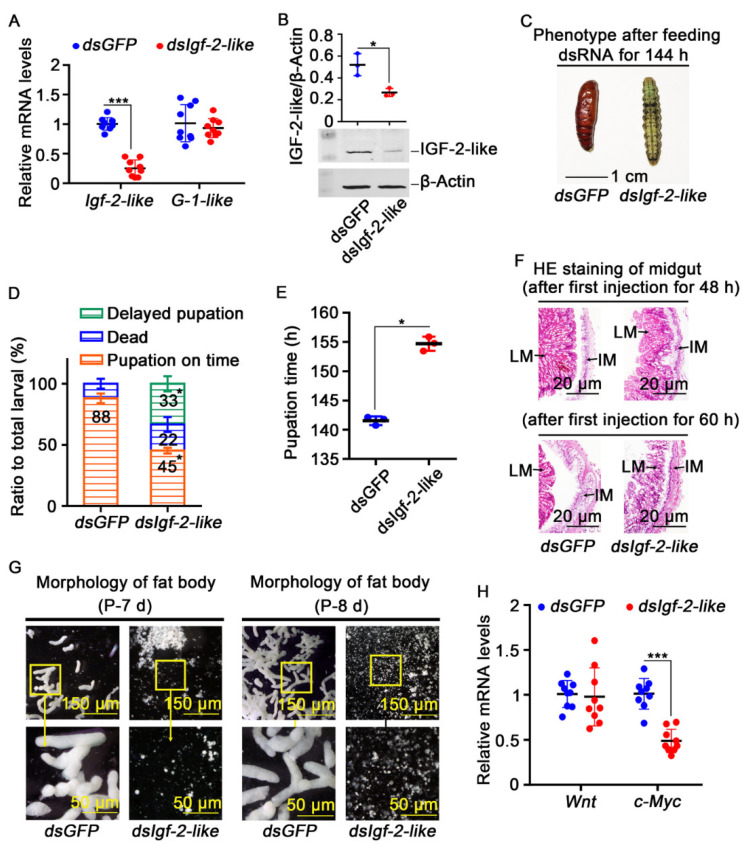
Knockdown *Igf-2-like* caused delayed pupation. (**A**) Detection of the knockdown efficiency of *dsIgf-2-like* by feeding the *E. coli*; the off-target effect was detected by *G-1-like*. (**B**) The fat body protein was extracted at 6th-126 h to detect the knockdown efficiency after *Igf-2-like* knockdown by injection of dsRNA. β-ACTB was used as an internal reference protein. Image J was used to calculate the ratio of IGF-2-like and β-ACTB band densities. (**C**) The phenotype of larvae 144 h after feeding of *dsIgf-2-like* and *dsGFP*. (**D**) Statistical analysis of the phenotype. The data for the phenotype ratio were collected in triplicate using thirty larvae each time by feeding the *E. coli*. The delayed pupation was defined as a pupation time longer than the average pupation time of the *dsGFP* (142 h) group by feeding the *E. coli*. (**E**) The pupation time by feeding the *E. coli*. (**F**) HE staining of the midgut after the injection of *dsIgf-2-like* and *dsGFP* for 48 h and 60 h. (**G**) Morphology of the fat body after the first injection for 10 d and 11 d by injection of dsRNA. (**H**) The expression levels of *Wnt* and *c-Myc* in the fat body were detected after *Igf-2-like* knockdown by injection of dsRNA. All the experiments comprised three biological repetitions. The error bar indicates the mean ± SD, and a *t*-test was used to show significant differences (* *p* < 0.05, *** *p* < 0.001).

To explore the function of IGF-2-like in imaginal midgut and fat body formation during metamorphosis, the expression of *Igf-2-like* was knocked down by feeding *E. coli* expressing dsRNA-targeting *Igf-2-like* in third stage larvae, or injecting dsRNA into 6th-72 h larva. The results showed that *Igf-2-like* was knocked down successfully and the pupation was delayed (Figure 6A–C). There was no significant difference in the average pupal weight between the *dsGFP* control group (0.41 g) and the *dsIgf-2-like* group (0.40 g). However, 33% of the larvae showed delayed pupation (Figure 6D). The average pupation time of the *dsIgf-2-like* group was 155 h and that of the *dsGFP* group was 142 h, i.e., the pupation time was delayed by about 13 h (Figure 6E). The growth of the imaginal midgut cells was repressed (Figure 6F). In addition, *dsIgf-2-like* repressed imaginal fat body formation at the pupal stage (Figure 6G). The mechanism of failure of imaginal fat body formation was analyzed by examining the expression levels of *Wnt* (AHN95659.1) and *c-Myc* (AHN95658.1), which are known to promote cell proliferation [35,36]. Knockdown of *Igf-2-like* resulted in decreased expression of *c-Myc*, but had no effect on *Wnt* in the fat body (Figure 6H). These results indicated that IGF-2-like promotes imaginal midgut and fat body growth and proliferation.

## 4. Discussion

We identified and revealed that the expression of IGF-2-like upon 20E stimulus is upregulated in the imaginal midgut and fat body cells during metamorphosis. IGF-2-like plays key roles to maintain the growth and proliferation of imaginal midgut and fat body cells. Interestingly, IGF-2-like-positive fat body cells did not undergo apoptosis and were identified as the progenitors of the adult fat body.

### 4.1. 20E Upregulates IGF-2-like Expression

IGF-2 plays important roles in fetal growth and development [6]. IGF-2 also plays roles in several cancers, such as breast, prostate and colorectal, by promoting cell growth and survival in humans [37]. IGF-2 promoted neural stem cell self-renewal via insulin receptor-A (IR-A) in cell culture studies [38]. However, little is known about the regulation mechanism of IGF-2 expression. High levels of 20E at the metamorphic stage promote programmed cell death in larval tissues and growth and division in developing imaginal tissues [39]. The 20E upregulated the expression of eight ILPs during metamorphosis in *H. armigera* [19]. The 20E also upregulates the expression of *Ilp4* and *Ilp5* in *Aedes aegypti* [40]. The 20E is found in insects and many plants, and has been shown to have possible clinical applications to treat neuromuscular, cardio-metabolic, and respiratory diseases [41].

DILP5 is highly expressed in Malpighian tubules and participates in the oxidative stress reaction [42]. DILP8 is expressed in imaginal discs to control insect size and development [17]. DILP2 is widely expressed in the whole imaginal discs and controls body size [43]. DILP6 is highly expressed in the fat body of the larval and imago stages, which promotes the growth of imaginal tissues [16]. In this study, IGF-2-like was clustered with DILP8 and human IGFs by phylogenetic analysis, and is highly expressed in the imaginal midgut and fat body cells at metamorphic stages under 20E regulation. The 20E represses insulin receptor expression during metamorphosis [19]; therefore, the possible receptor of IGF-2-like in insects, IGF-2/M6P receptor, or the receptor tyrosine kinase, needs to be studied in future work.

In addition to the fat body and midgut, IGF-2-like is also expressed in the epidermis and brain at the feeding stage; therefore, it might play a pro-growth role in the epidermis and brain during larval development. The roles of IGF-2-like and regulatory hormones at the feeding stage require further study. IGF-2-like has a signal peptide; however, no bands were detected from the hemolymph, probably because the hemolymph was diluted at 1:8 in PBS in our experiment. Another reason is IGF-2-like can be degraded by insulin-degrading enzymes [44]. IGF-2-like was not observed to be cleaved in our study according to the molecular mass analysis.

### 4.2. IGF-2-like Promotes Cell Growth and Proliferation

The larval midgut and fat body are degraded by autophagy and apoptosis during metamorphosis, whereas the imaginal midgut and fat body form via cell growth and proliferation [45]. Cell proliferation occurs during the formation of the adult fat body of *H. armigera*, although endoreplication is the primary mechanism. We revealed here that IGF-2-like expressed in the imaginal midgut and fat body cells promoted the growth and proliferation of the imaginal midgut and fat body growth, which protects them from cell death.

We found that IGF-2-like promoted the cell proliferation of the imaginal midgut and fat body through c-Myc. c-Myc is an oncogene involved in cell proliferation. Overexpression of c-Myc would change the expression of a series of related genes, such as increasing the levels and activities of snail and vimentin, and reducing the level and activity of E-cadherin, thus leading to low expression of the tumor suppressor gene *Chibby*, which leads to laryngeal squamous cell carcinoma [46]. Oncogenes and growth factors can promote cell proliferation, among which IGF-1 and c-Myc are used as targeted factors for cancer treatment [47].

### 4.3. The IGF-2-like-Positive Fat Body Cells Are the Progenitors of the Adult Fat Body

The fat body undergoes autophagy and apoptosis at the 6th-96 h stage; however, the adult fat body proliferates and grows markedly in late pupal stages in *H. armigera* [29]. The larval fat body and the adult fat body are not distinguished during fat body remodeling at earlier metamorphic stages, and the mechanism of adult fat body formation was unclear. Although the adult fat body is hypothesized to originate from the regeneration of a part of the remaining larval fat body [24], or by the differentiation of imaginal progenitor cells [25], no regenerative larval cells or imaginal progenitor cells have been observed. In this study, we provided evidence that IGF-2-like positive cells are the progenitor cell population that forms the adult fat body. This is supported by our finding that IGF-2-like cells spread among some fat body cells, which were not co-localized with apoptotic signals, as revealed by activated CASP3 staining; in addition, knockdown of *Igf-2-like* repressed adult fat body formation.

## 5. Conclusions

**Figure 7:**
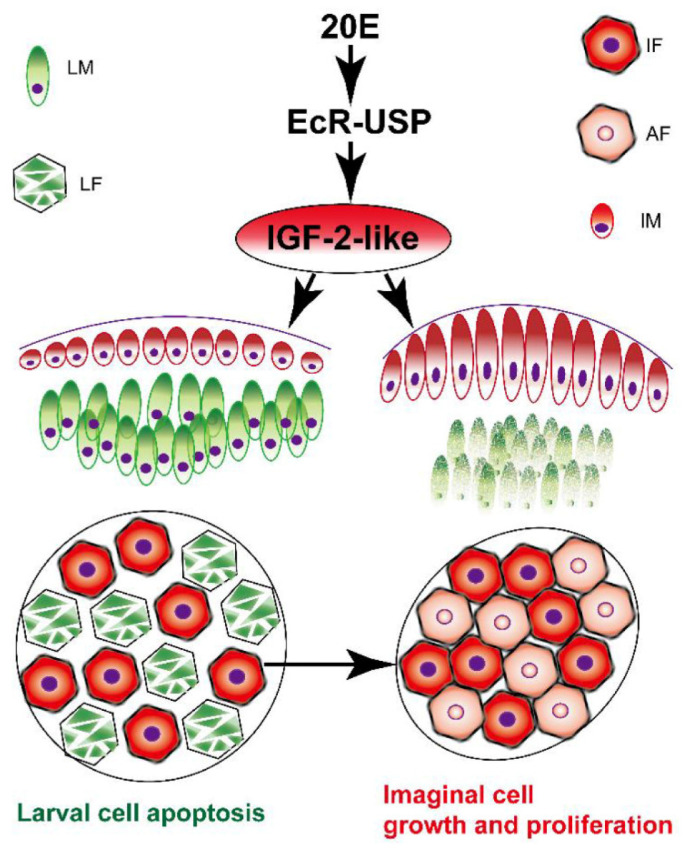
Schematic illustration of the mechanism by which IGF-2-like functions to determine cell proliferative fate. The 20E increases the expression of *Igf-2-like* via the nuclear receptor EcR. IGF-2-like localizes to the imaginal midgut and fat body to promote imaginal cell growth and proliferation. The larval cells that do not express IGF-2-like die via programmed cell death. LM: larval midgut cells. LF: larval fat body cells. IF: imaginal fat body cells (progenitors of adult fat body cells). AF: adult fat body cells proliferated from the progenitor of adult fat body cells. IM: imaginal midgut. Red indicates IGF-2-like expression. Green indicates CASP3 expression.

The 20E upregulated the expression of IGF-2-like in the imaginal midgut and fat body during tissue remodeling. IGF-2-like promotes imaginal midgut and fat body growth and proliferation, thereby avoiding imaginal midgut and fat body death. IGF-2-like marks progenitor cells of the imaginal fat body that are mixed among the larval fat body cells (Figure 7). It will be interesting for future studies to determine whether steroid hormone regulation of insulin ligands in a conserved phenomenon.

## Data Availability

All data are contained within the article.

## Supplementary Materials

The following supporting information can be downloaded at: https://www.mdpi.com/article/10.3390/cells11111799/s1, Figure S1: MAGE 5 analysis of the phylogenetic tree of ILPs in *B. mori, D. melanogaster and H. sapiens.*; Figure S2: Antibody specific detection of IGF-2-like in the 6th-96 h fat body. Figure S3: Detection of antigen specificity; Figure S4: Expression profiles of IGF-2-like in the epidermis and brain. Figure S5: The number of nuclei in IGF-2-like-positive fat body cells stained by DAPI in the fat body at 6th-24 h and 6th-120 h. Figure S6. IGF-2-like, pHH3 and CASP3 were not detected in the midgut and fat body at the 6th-24 h by immunofluorescence. Table S1: Primers used in the experiments.

## Abbreviations

ILP	insulin-like peptide;
IGF	insulin-like growth factor;
20E	20-hydroxyecdysone;
CASP3	caspase 3;
pHH3	phosphorylated histone H3;
HE staining	hematoxylin and eosin staining.
