# Supplementary material for: Insulin-like Growth Factor 2 Promotes Tissue-Specific Cell Growth, Proliferation and Survival during Development of Helicoverpa armigera
Source: Cells. 2022 May 31;11(11):1799. doi: 10.3390/cells11111799 (PMC9180042; doi:10.3390/cells11111799)
Supplement: Supplementary file 1 [file cells-11-01799-s001.zip › cells-1732610-supplementary.pdf]

Supplementary Figure and Figure legends

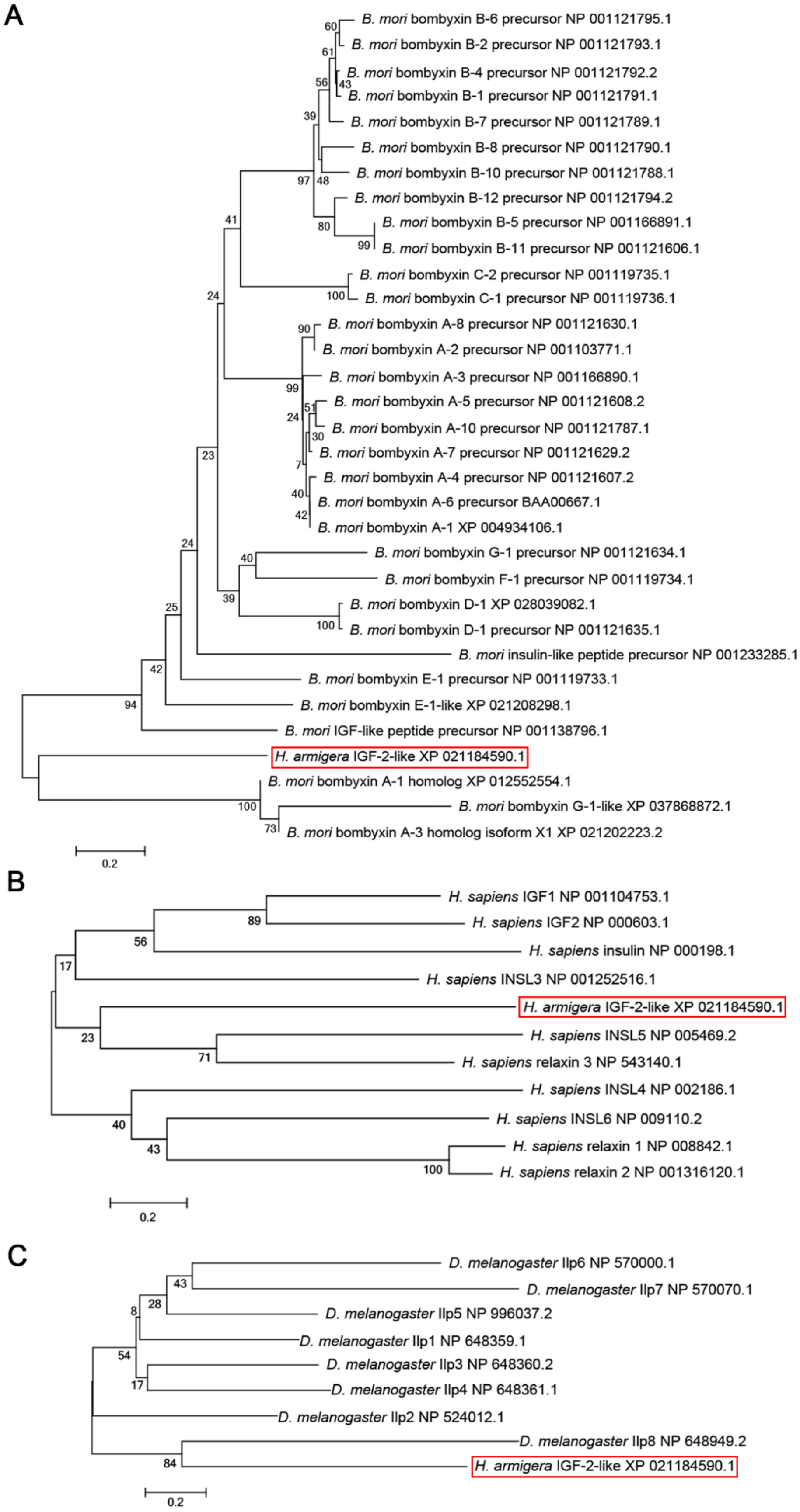

**Figure S1. MAGE 5 analysis of the phylogenetic tree of ILPs in *B. mori*, *D. melanogaster*, and *H. sapiens*.** **(A)** Phylogenetic tree of *B. mori* ILPs. **(B)** Phylogenetic tree of ILPs in *D. melanogaster*. **(C)** Phylogenetic tree of *H. sapiens* ILPs. The red box denotes IGF-2-like.

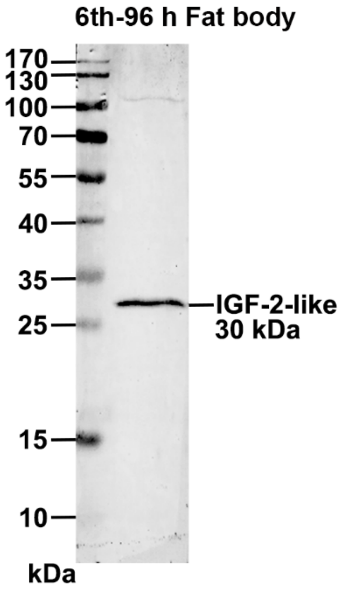


**Figure S2.** Antibody specific detection of IGF-2-like in the 6th instar larval (6th-96 h) fat body.

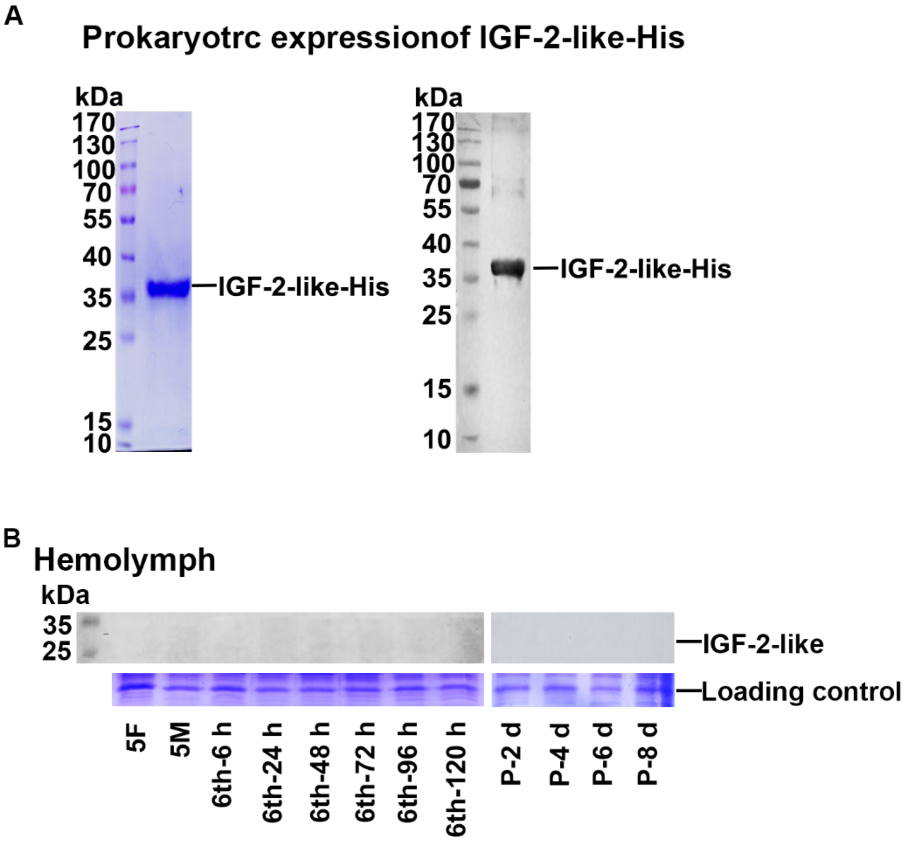


**Figure S3.** **(A)** SDS-PAGE (left) and western blotting (right) were used to detect the recombinant of IGF-2-like-His overexpressed in *E. coli* from plasmid pET-30a. The gel concentration was 12.5%. **(B)** The protein level of IGF-2-like in the hemolymph at different stages.

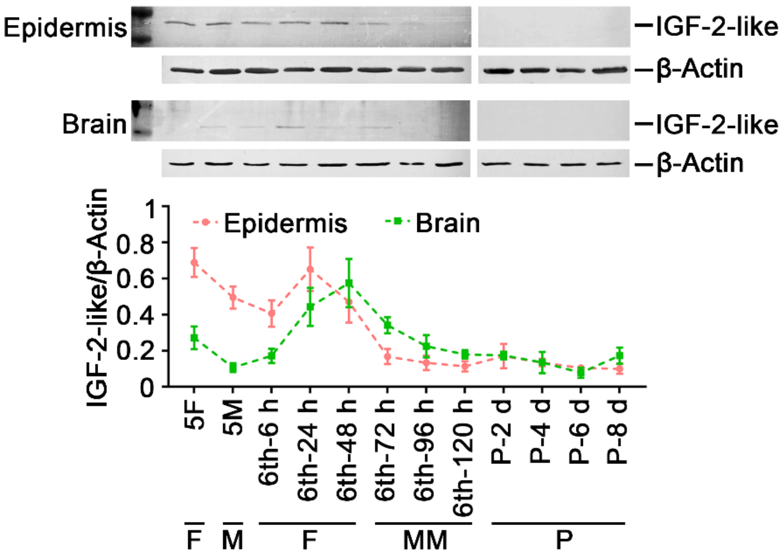


**Figure S4. Expression profiles of IGF-2-like in the epidermis and brain.** ACTB was used as an internal reference protein. The concentration of the gel was 12.5%. Image J was used to calculate the ratio of IGF-2-like and ACTB band densities. All the experiments were performed using three biological replicates. The error bar indicates the mean ± SD. 5F: the 5th instar feeding stage; 5M: the 5th molting stage; 6th-6 h, 6th-24 h, 6th-48 h, 6th-72 h, 6th-96 h, and 6th-120 h correspond to different stages of the 6th instar; P-2 d, P-4 d, P-6 d, and p-8 d correspond to different pupal stage. F: feeding stage; M: molting stage; MM: metamorphic molting stage; P: pupal stage.

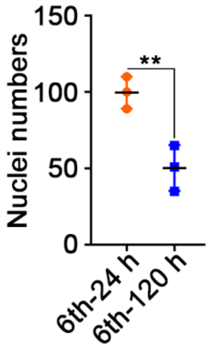


**Figure S5.** The number of nuclei in IGF-2-like-positive fat body cells stained by DAPI in the fat body at 6th-24 h and 6th-120 h.

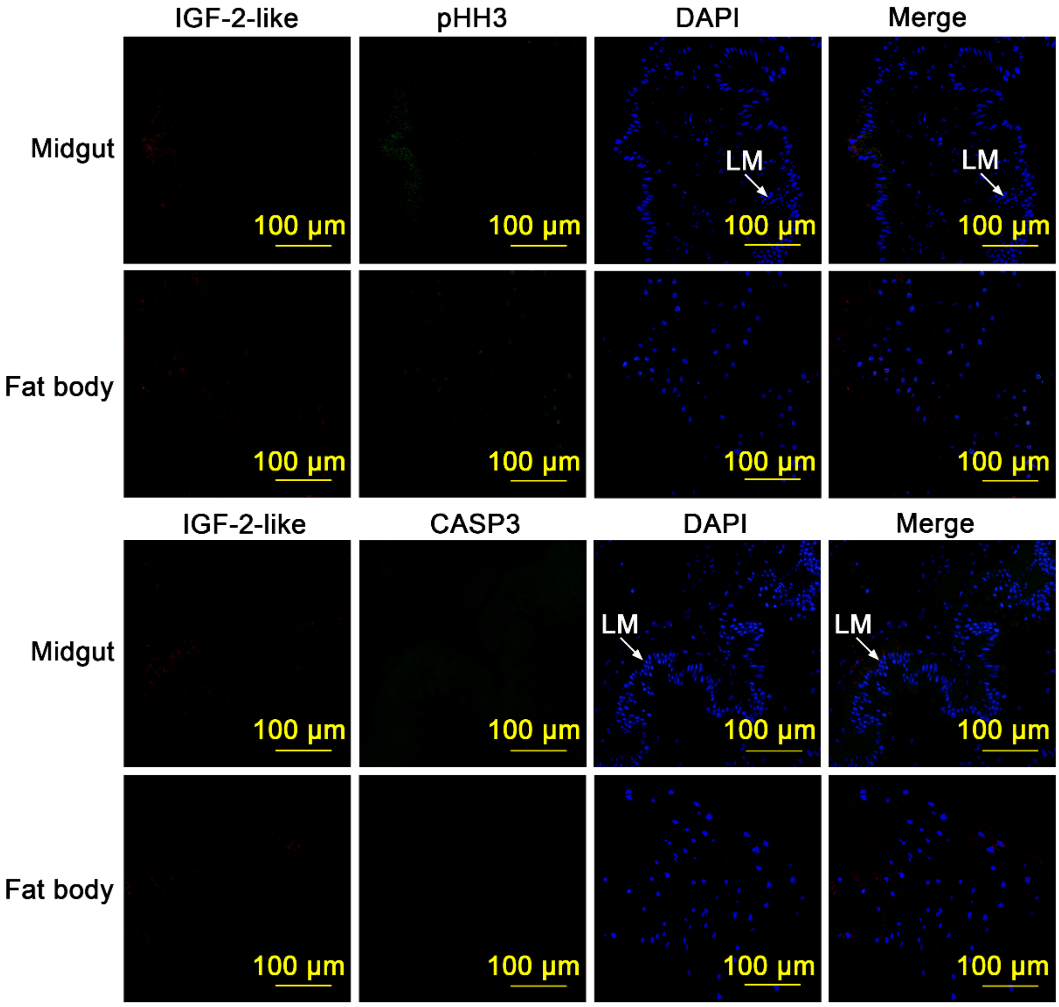


**Figure S6.** IGF-2-like, pHH3, and CASP3 were not detected in the midgut and fat body at the 6th-24 h by immunofluorescence. The scale bar is 100 μm.

Table S1 Primers used in the experiments

Exp-Igf-2-like-F tactcagatatcatgttagtaaacaaggca

Exp-Igf-2-like-R tactcagagctctgcgtagacgacgtacgc

Igf-2-like-RTF ctgccacgcttacaacagtcc

Igf-2-like-RTRtcagcaataccgtttcccact

Igf-2-like-RNAiF gcgtaatacgactcactataggacgacaagtgggaaacgg

Igf-2-like-RNAiR gcgtaatacgactcactataggaggtcagtgttgagtgtccg

Igf-2-like-swRNAiF tactcactgcagacgacaagtgggaaacgg

Igf-2-like-swRNAiR tactcactcgagaggtcagtgttgagtgtccg

GFP-RNAiF gcgtaatacgactcactatagggatggtcccaattctcgtggaac

| | |
|---|---|
| GFP-RNAiR | gcgtaatacgactcactatagggacttgaagttgaccttgatgcc |
| GFP-swRNAiF | tactcagcggccgctggtcccaattctcgtggaac |
| GFP-swRNAiR | tactcactcgagcttgaagttgaccttgatgcc |
| G-1-like-RTF | gttgacggaaatgaaagg |
| G-1-like-RTR | cagacggtaaatcactaatc |
| c-Myc-RTF | atgaagactggaggataatgga |
| c-Myc-RTR | tgttgtgggtgtagtggttg |
| Wnt-RTF | gtcacggtatgtcaggctcg |
| Wnt-RTR | ggctccaggtcagtattaggc |
| β-actin-RTF | cctggtattgctgaccgtatgc |
| β-actin-RTR | ctgttggaaggtggagagggaa |
